# Endoscopic Treatment as the Rescue Therapy for Recurrent Bleeding after Transjugular Intrahepatic Portosystemic Shunt (TIPS)

**DOI:** 10.1155/2021/6627837

**Published:** 2021-08-02

**Authors:** Liyuan Ni, Xiaoquan Huang, Siyu Jiang, Lili Ma, Jianjun Luo, Shiyao Chen

**Affiliations:** ^1^Department of Gastroenterology and Hepatology, Zhongshan Hospital, Fudan University, 200032 Shanghai, China; ^2^Endoscopy Center and Endoscopy Research Institute, Zhongshan Hospital, Fudan University, 200032 Shanghai, China; ^3^Department of Interventional Radiology, Zhongshan Hospital, Fudan University, 200032 Shanghai, China

## Abstract

**Background:**

Transjugular intrahepatic portosystemic shunt (TIPS) is suggested as the salvage therapy for gastroesophageal variceal bleeding in cirrhosis. However, rebleeding might occur in some patients after TIPS. Currently, there is a lack of evidence in the endoscopic management of recurrent bleeding in these patients.

**Aims:**

To evaluate the efficacy of endoscopic treatment in cirrhotic patients with recurrent bleeding after TIPS.

**Methods:**

Cirrhotic patients with gastroesophageal varices who received endoscopic treatment for recurrent bleeding after TIPS were included.

**Results:**

6 patients were enrolled in this study. The median age of the patients was 47 years (range 27 to 65 years), and the duration of follow-up time was 346 (17-773) days. Stent stenosis or occlusion was found in 5 out of 6 patients after TIPS. Salvage endoscopic treatment, including esophageal variceal ligation (EVL), gastric variceal cyanoacrylate injection, esophageal variceal sclerotherapy, and balloon-occluded retrograde transvenous obliteration- (BRTO-) assisted endoscopic cyanoacrylate injection. Among included patients, 2 died shortly after EVL (14 and 19 days) due to variceal bleeding. Among other 4 patients, 2 had rebleeding episodes at 422 and 789 days, respectively.

**Conclusion:**

Endoscopic treatment may be an option for recurrent bleeding after TIPS in selected patients. Further studies are needed to carefully define the indication and efficacy of this option.

## 1. Introduction

Portal hypertension (PHT) is a clinical syndrome mostly caused by liver cirrhosis. Other etiologies, such as isolated portal vein thrombosis and Budd-Chiari syndrome, account for less than 10% of the cases [1]. Portal hypertension is identified when the portosystemic pressure gradient is equal to or above 6 mmHg. Increasing portal pressure generates hemodynamic abnormality and portosystemic collateral formation, leading to a series of complications, which may include varices, ascites, hepatic encephalopathy (HE), infection, and even death. Variceal bleeding is among the most common, refractory, and life-threatening complications of PHT. Even with intervention, the 1-year rebleeding rate reaches 60% and mortality 33% [2]. Current guidelines suggest that nonselective beta-blockers (NSBBs) plus endoscopic variceal ligation (EVL) are a standard therapy for secondary prophylaxis of variceal rebleeding [3]. Transjugular intrahepatic portosystemic shunt (TIPS) is recommended as the rescue therapy after the failure of combined medical and endoscopic therapies for prophylaxis of variceal bleeding. In recent years, early TIPS, defined as performing TIPS within 72 h (ideally <24 h), is also recommended as the preemptive therapy for patients at high risk of treatment failure (e.g., Child − Pugh class C < 14 points, Child-Pugh class B with active bleeding) [4, 5].

TIPS uses a radiologically inserted stent to divert blood from the portal vein to the hepatic vein, thus effectively reducing portal pressure. One meta-analysis comparing TIPS to EVL plus NSBB showed that TIPS remarkably reduced the rebleeding rate, from 41.7% to 15.4% (OR = 0.27; 95% CI, 0.19–0.39; *P* < 0.00001), without improving overall survival (OR = 0.84; 95% CI, 0.63–1.12; *P* = 0.23) [6]. In recent years, covered stent graft such as polytetrafluoroethylene- (PTFE-) covered stent has been developed to improve the stent patency. Triantafyllou et al. concluded that the 1-year primary patency of stent rose from 52% to 84% by using covered stent instead of the bare stent, while rebleeding rate dropped from 18% to 9% [7]. In clinical practice, patients at high risk, NSBB nonresponder, and patients with extraluminal collaterals prefer to receive TIPS treatment first since it has been established that endoscopic treatments are less effective in these patients [8–11].

The artificial shunt created by TIPS also causes some complications, such as hepatic encephalopathy (HE), shunt dysfunction, and cardiac volume overload [12, 13]. As the most common complication of TIPS, the incidence of HE is estimated to range from 17.6 to 42.8% [14–17]. For patients with HE history, stent revision might increase HE episodes' risk because of the portosystemic shunt extension [18]. For patients with recurrent variceal bleeding after TIPS and those unable to have shunt recanalization, endoscopic treatment's efficacy and safety remain unclear. The aim of this study was to evaluate the efficacy and safety of endoscopic treatment for preventing recurrent variceal bleeding after TIPS.

## 2. Materials and Methods

### 2.1. Study Design

This retrospective observational study was conducted in Zhongshan Hospital, Fudan University (Shanghai, China), between January 2017 and August 2019. Patients fulfilling the following criteria were included: (1) a history of variceal bleeding and received TIPS, (2) rebleeding after TIPS, (3) had TIPS revision and re-TIPS technically impossible, and (4) received endoscopic treatment for recurrent esophageal and/or gastric variceal bleeding after TIPS. Patients combined with hepatocellular carcinoma and other deadly diseases were excluded. All the enrolled patients were followed up by phone until 31^st^ July 2020 or death. The study was approved by the Ethics Committee of Zhongshan Hospital, Fudan University.

### 2.2. Endoscopic Treatments

All the patients underwent elective endoscopic treatment, no vasoactive drugs or preventative antibiotics were used periendoscopic management at our center. Endoscopic treatments were performed by experienced endoscopists according to guidelines and our published study [19, 20]. Esophageal varices were treated with endoscopic variceal ligation using 6-band ligators (Cook Endoscopy, Winston-Salem, North Carolina, USA) at 1 cm above the Z-line in a spirally ascending way under endoscopy (Olympus, Tokyo, Japan). Endoscopic cyanoacrylate injection or BRTO-assisted endoscopic cyanoacrylate injection (E-BRTO) was used for gastric varices. In endoscopic cyanoacrylate injection, the sandwich method defined as the injection of lauromacrogol (Tianyu Pharmaceutical, Zhejiang, China)-cyanoacrylate (Compant, Beijing, China)-lauromacrogol through a needle (NM-400L-0421; Olympus, Tokyo, Japan) was applied to multiple points to ensure complete varices obturation. E-BRTO was performed by inserting a balloon catheter (Boston Scientific, Marlborough, Massachusetts, USA) to the gastrorenal or gastrosplenorenal shunt to occlude blood flow in it, followed by the endoscopic cyanoacrylate injection of gastric varices and esophageal variceal ligation.

### 2.3. Primary and Secondary Outcomes

The primary outcome of the study was the incidence of rebleeding after endoscopic treatment. Rebleeding was defined as all-cause hemorrhage from the gastrointestinal tract leading to hematemesis, hematochezia, or melena. The secondary outcomes were 1-year mortality rate and adverse events during the follow-up period. In-hospital data were collected from electronic medical records. Follow-up time was measured from the date of first post-TIPS endoscopic treatment and the loss of follow-up or 31^st^ July 2020.

### 2.4. Statistical Analysis

Statistical analysis was performed using SPSS 25.0 software (SPSS Inc., Chicago, Illinois, USA). Continuous variables are reported as mean with standard deviation (SD) for normally distributed values and median with interquartile range (IQR) when the values' distribution significantly deviated from the normal distribution. Categorical variables are described as the frequency with percentage.

## 3. Results

### 3.1. Demographics

Among patients admitted to Zhongshan Hospital between January 2017 and August 2019 who received endoscopic treatments such as secondary prophylaxis for variceal bleeding, 76 patients received both endotherapies and TIPS. After reviewing their medical records and excluding patients who only received endotherapies before TIPS, 6 patients were finally enrolled in this study.

**Figure 1:**
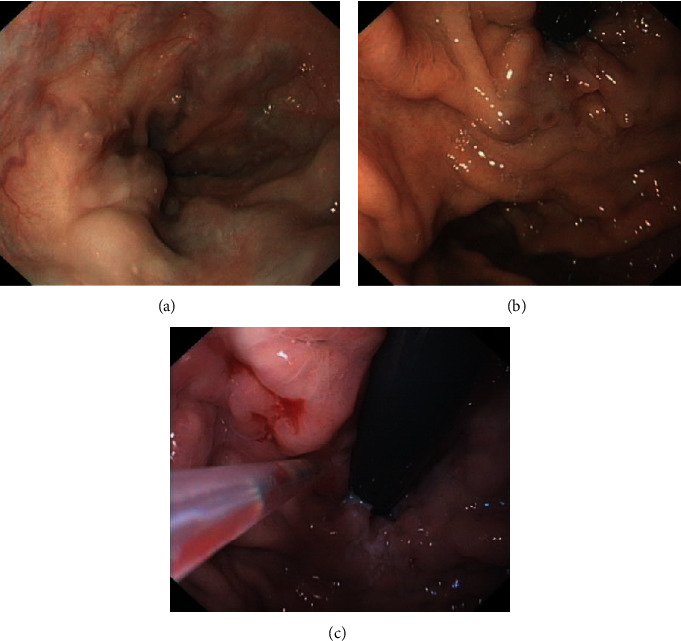
Endoscopic finding of (a) esophageal varices, (b) gastric varices, and (c) intravascular injection of cyanoacrylate of neovascularisation of gastric varices.

Clinical characteristics of these 6 patients are shown in Table 1. They included 5 male and 1 female patients with a median age of 47 years (range, 27 to 65 years). The etiology of portal hypertension consisted of HBV (*n* = 1), alcoholic (*n* = 2), autoimmune (*n* = 1), schistosomiasis (*n* = 1), and Budd-Chiari syndrome (*n* = 1). 4 patients had portal vein thrombosis (PVT). All the included patients were confirmed with severe esophageal and/or gastric varices by endoscopy (Figure 1). Gastroesophageal varices type 2 (GOV2) was the most common type of varices (50%), while 1 patient had gastroesophageal varices type 1 (GOV1) and 2 had esophageal varices (EV). Endoscopic treatments were performed for at least one time, 2 with EVL alone, 1 with BRTO-assisted endoscopic cyanoacrylate injection, and 3 with EVL plus gastric variceal cyanoacrylate injection. Notably, the patient who received BRTO-assisted endoscopic cyanoacrylate injection had been previously treated with esophageal variceal sclerotherapy (EVS) twice after TIPS at another hospital; however, these treatments failed to prevent rebleeding. After endotherapy, 2 patients continually took NSBB during the follow-up period. The median follow-up time was 346 (17-773) days.

### 3.2. TIPS Procedure

The specific information of previous TIPS is shown in Table 2. The indications of TIPS were high hepatic venous pressure gradient (HVPG) (over 20 mmHg), rebleeding after endotherapies, Budd-Chiari syndrome, and the existence of extraluminal collaterals. Four patients (#1-3 and #5) undergoing TIPS at our center were inserted with 2 stents, including one 8 mm PTFE-covered stent plus one 10 mm bare stent. The average portosystemic pressure gradient reduction after stent placement was 8 ± 3.46 mmHg. 5 patients had shunt dysfunction (#1 and #5 had stent stenosis, and #3-4 and #6 had stent occlusion) after TIPS. Among them, 3 patients (#1, #3, and #6) received stent revision at least once. 2 patients experienced HE shortly after TIPS (#6 in 26 days and #5 in 79 days). All of them experienced rebleeding episodes after TIPS with the median rebleeding-free time of 367 (161.8-660.0) days.

### 3.3. Outcomes of Endoscopic Treatments

**Figure 2:**
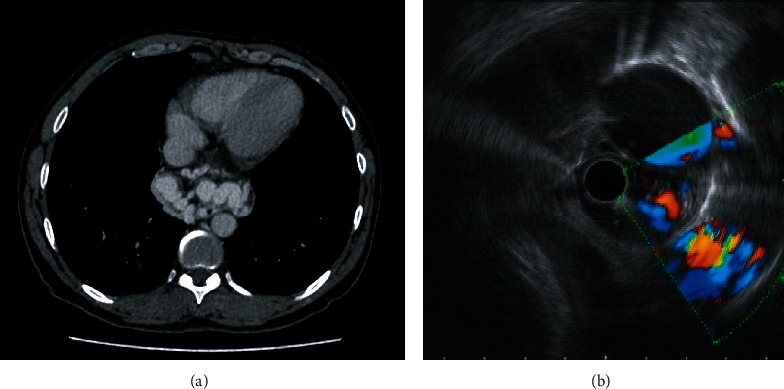
CT and endoscopic ultrasonography(EUS) findings of extraluminal collaterals. (a) Large paraesophageal veins on CT (patient #1). (b) Large paragastric veins on EUS (patient #5).

Four patients (#1-2, and #5-6) experienced rebleeding after endoscopic treatments. The median rebleeding-free time was 391 (96.8-673.3) days. 2 patients (#1 and #5) with paraesophageal veins or paragastric veins (Figure 2) suffered from hemorrhage shortly after EVL (in 7 and 9 days) and quickly died (14 and 18 days after EVL) due to mass blood loss. The other two patients (#2 and #6) went through one episode of rebleeding in 422 and 789 days, respectively (Table 3). The 1-year rebleeding rate was 33.3% (2 in 6). Patients were divided into two groups by Child-Pugh class, Class A+B (*n* = 3) and Class C (*n* = 3); the 1-year rebleeding rate was not different between groups (both 33.3%).

The 1-year survival rate and the overall survival rate was 66.7% and 50%, respectively. The median survival time of six patients was 558.5 (100.5-883) days. One patient (#6) who was diagnosed with hepatocellular carcinoma during the follow-up died of gastrointestinal hemorrhage 792 days after BRTO-assisted endoscopic cyanoacrylate injection.

## 4. Discussion

Endotherapies and TIPS are both effective ways of treating esophageal, gastric varices. Endotherapies are local treatments that block the blood flow in varices by ligation or cyanoacrylate injection, while TIPS significantly reduces the blood flow in varices and prevents rupture. TIPS is generally considered a more advanced treatment compared to endotherapies, but it is still not completely effective. HE is the most common complication, but stent type and operators' skills also affect the outcomes of TIPS [21]. Rebleeding mostly occurs because of the stent stenosis and occlusion [7]. Thus, a commonly used therapy for such cases is TIPS revision. Yet, some patients might not benefit from stent revision. For patients suffering from bleeding after TIPS without shunt dysfunction [22, 23], TIPS revision is not effective. Some patients may also bleed again after TIPS revision, which implies that TIPS revision is not effective in preventing variceal rebleeding. Moreover, if a patient has HE after TIPS, stent revision may be a dangerous option [24]. For these patients, endoscopic treatments should be considered as they have been associated with smaller risk, less complication, and lower cost. To the best of our knowledge, this is the first study that examined the outcomes of endoscopic treatment after TIPS failure. All the patients who received endoscopic treatment for variceal bleeding at our center between January 2017 and August 2019 were screened, and their medical records were checked for previous treatment history. Finally, 6 eligible patients were enrolled; 2 did not experience any gastrointestinal hemorrhage during follow-up, 2 had only one episode of re-bleeding with re-bleeding-free time over 1 year, and 1was diagnosed with hepatocellular carcinoma. These results showed that endoscopic treatment could be helpful in preventing rebleeding of patients that might not be suitable to receive TIPS revision.

In this study, one patient (#3) with Budd-Chiari syndrome (BCS) underwent TIPS revision for 4 times but still experienced rebleeding. Then, he received EVL+gastric variceal cyanoacrylate injection, which stopped bleeding before the endpoint. BCS is a rare disease defined by hepatic venous outflow tract obstruction. According to the current guideline, TIPS is indicated when severe portal hypertension complications exist, while endoscopic treatment is not included in the recommended stepwise therapies [25]. In a literature review, stent occlusion tends to be more frequent in BCS patients because of their prothrombotic states. As BCS patients in this study experienced no more rebleeding, endoscopic treatment could be an effective choice for treating BCS-related variceal bleeding. Also, if patients repeatedly experience stent occlusion after TIPS and TIPS revision, especially those combined with portal vein thrombosis, this suggests that these patients are in hemostatic imbalance, and they might benefit from endoscopic treatments.

We observed that one patient (#6) who had HE after TIPS received both E-BRTO and EVL. She had a 789-day period free of rebleeding. HE is a major complication of TIPS. The general prevalence of overt hepatic encephalopathy (OHE) at the diagnosis of cirrhosis is 10%-14%. After TIPS, the median cumulative 1-year incidence of OHE reaches 10%-50% [26], which is related to the portosystemic shunt that brings ammonia to circulation without metabolism. Stent extensions are not appropriate for these patients, so endoscopic treatment might be suitable for them. E-BRTO is a newly developed technology used to treat gastric varices. Instead of injecting sclerosant like traditional BRTO that tends to increase portal resistance and worsen GOV in the long-term, cyanoacrylate is injected at targeted varices under endoscopy [27]. This is also convenient for patients in need of EVL to deal with esophageal varices.

In the present study, only two patients with paraesophageal veins or paragastric veins both experienced bleeding quickly after EVL and died within 20 days. This suggested that patients with extraluminal collaterals were much more likely to obtain bad results after endotherapies than other patients in this situation, which is also consistent with a few studies that focused on examining whether the presence of extraluminal collaterals is a predictor related to poor prognosis of endoscopic treatments. A prospective cohort study of esophageal varices found that large periesophageal collateral veins and perforating veins were independent risk factors of esophageal varices recurrence (*P* < 0.0001, *P* < 0.001) [28]. In another retrospective study that enrolled 170 patients, the existence of paragastric, gastric perforating, and esophageal perforating veins was significantly associated with poor patient response to endoscopic treatments (*P* < 0.001), which was defined as variceal rebleeding, Grade 3 (F3 or diameter > 5 mm) varices or obvious red wale marks under endoscopic examination [11]. Thus, we hypothesized that endoscopic treatments after TIPS failure are only suitable for patients without extraluminal collaterals.

The present study has some limitations. First, this was a single-center study, which definitely causes selection bias. Second, the sample size was small because of rare endotherapy use after TIPS, thus making the analysis hardly significant. Large multicenter prospective clinical researches are required for a better evaluation of endoscopic treatment after TIPS failure in the future.

In conclusion, endoscopic treatment might be helpful to prevent rebleeding after TIPS. Patients having a history of HE, bleeding without stent stenosis or occlusion, and bleeding after TIPS revision or without extraluminal collaterals might be suitable candidates for endoscopy.

## Figures and Tables

**Table 1:** Baseline characteristics of the patients.

Patients	
Sex, *n* (%)	
Male	5 (83.3)
Female	1 (16.7)
Age (mean ± SD)	47.5 ± 13.6
Etiology, *n* (%)	
Alcoholic	2 (33.3)
Autoimmune	1 (16.7)
Budd-Chiari syndrome	1 (16.7)
Hepatitis B virus	1 (16.7)
Schistosomiasis	1 (16.7)
Varices classification, *n* (%)	
GOV1	1 (16.7)
GOV2	3 (50.0)
EV	2 (33.3)
PVT, *n* (%)	4 (66.7)
Ascites, *n* (%)	5 (83.3)
Hb (mean ± SD)	83.3 ± 27.8
ALT (mean ± SD)	25.5 ± 18.9
AST (mean ± SD)	52.2 ± 43.3
Child-Pugh score (mean ± SD)	9 ± 2
Child-Pugh class, *n* (%)	
Class A	2 (33.3)
Class B	1 (16.7)
Class C	3 (50.0)
Hypertension, *n* (%)	1 (16.7)
Diabetes, *n* (%)	2 (33.3)
HE history, *n* (%)	2 (33.3)
Use of NSBB, *n* (%)	1 (16.7)
Use of anticoagulants, *n* (%)	6 (100.0)

SD: standard deviation; PVT: portal vein thrombosis; Hb: hemoglobin; ALT: alanine aminotransferase; AST: aspartate aminotransferase; HE: hepatic encephalopathy; NSBB: nonselective beta-blockers.

**Table 2:** Detail of previous TIPS treatments.

Patient	Endoscopic sessions before TIPS	HE before TIPS	Pre-TIPS HVPG (mmHg)	Extraluminal collaterals	Stent type	Pre-TIPS gradient (mmHg)	Post-TIPS gradient (mmHg)	Stent stenosis/occlusion	Times of TIPS revision	HE after TIPS	Post-TIPS rebleeding-free time (d)
1	0	Absent	23	Absent	8 mm covered stent+10 mm bare stent	29	22	Stenosis	2	Absent	171
2	1	Absent	20	Absent	8 mm covered stent+11 mm bare stent	33	20	Absent	0	Absent	280
3	0	Absent	NA	Absent	8 mm covered stent+12 mm bare stent	29	24	Occlusion	4	Absent	459
4	0	Absent	NA	Absent	Unknown	NA	NA	Occlusion	0	Absent	454
5	0	Absent	NA	Present	8 mm covered stent+14 mm bare stent	21	14	Stenosis	0	Present	1263
6	1	Absent	NA	Present	Unknown	NA	NA	Occlusion	1	Present	134

HVPG: hepatic venous pressure gradient; NA: not available; HE: hepatic encephalopathy.

**Table 3:** Detail of endoscopic treatments and outcomes.

Patient	Child-Pugh classification	Endoscopic treatment	Use of NSBB during follow-up	Rebleeding	Time interval of rebleeding (d)	Death	Time interval of death (d)	Cause of death
1	C	EVL	None	Present	7	Present	14	Rebleeding
2	C	EVL+gastric variceal cyanoacrylate injection	Carvedilol	Present	422	Absent	/	/
3	C	EVL+gastric variceal cyanoacrylate injection	Propranolol	Absent	/	Absent	/	/
4	A	EVL+gastric variceal cyanoacrylate injection	None	Absent	/	Absent	/	/
5	B	EVL	None	Present	9	Present	18	Rebleeding
6	A	BRTO-assisted endoscopic cyanoacrylate injection	None	Present	789	Present	792	Rebleeding

EVL: endoscopic variceal ligation; BRTO: balloon-occluded retrograde transvenous obliteration.

## Data Availability

The datasets used and analyzed during the current study are available from the corresponding author on reasonable request.
